# Genome Sequencing of the Antibiotic-Resistant *Leucobacter* sp. HNU-1 and Its Developmental Toxicity in *Caenorhabditis elegans*

**DOI:** 10.3390/ijms26083673

**Published:** 2025-04-13

**Authors:** Jiaming Ju, Xinhe Lu, Ziqing Gao, Hongyan Yin, Shunqing Xu, Hanzeng Li

**Affiliations:** 1 School of Life and Health Sciences, Hainan University, Haikou 570228, China; lyeming@hainanu.edu.cn (J.J.); 23220860010016@hainanu.edu.cn (Z.G.); 2 School of Tropical Agriculture and Forestry, Hainan University, Haikou 570228, China; yinhy@hainanu.edu.cn; 3 School of Environmental Science and Engineering, Hainan University, Haikou 570228, China

**Keywords:** *Caenorhabditis elegans*, host–microbe interaction, multidrug resistance, diapause induction

## Abstract

To date, *Leucobacter* species have been identified from diverse sources with various ecological and functional roles. However, the genomic features and pathogenic potential of antibiotic-resistant *Leucobacter* strains remain understudied. Here, we isolated the *Leucobacter* sp. HNU-1 from tropical Hainan Province, China, and found it can induce diapause in *Caenorhabditis elegans* following ingestion, while exhibiting no significant effects on the nematode’s lifespan, survival rate, locomotion, and intestinal epithelial cells. This bacterium demonstrates resistance to multiple antibiotics, including kanamycin, streptomycin, sulfonamides, and vancomycin. On LB medium, *Leucobacter* sp. HNU-1 forms yellow, opaque colonies with a smooth, moist surface, regular edges, a convex center, and no surrounding halo, with diameters ranging from 2 to 3 mm. Furthermore, we performed whole-genome sequencing using third-generation high-throughput sequencing technology. De novo assembly revealed a genome size of 3,375,033 bp, with a GC content of 70.37%. A total of 3270 functional genes, accounting for 88.98% of the genome, were annotated, along with six potential CRISPR sequences and other genetic elements. Genomic and bioinformatic analyses further identified antibiotics-related genes. This research provides a theoretical foundation for investigating antibiotic-resistant environmental bacteria in tropical environments and offers new insights into potential therapeutic strategies for microbial infections and host–microbe interactions.

## 1. Introduction

The taxonomic position of the genus *Leucobacter* was formally established in 1996 [1]. Its naming and classification were grounded in comprehensive phylogenetic studies, with particular emphasis on analyses of 16S rRNA gene sequences and cellular chemical characteristics. Prior to this formal establishment, these bacteria were classified among other related Gram-positive bacteria with high G+C content. However, molecular phylogenetic studies revealed distinct taxonomic features that rendered them unsuitable for inclusion in their original taxonomic groups. Consequently, Takeuchi and Hatano [1] proposed the creation of the new genus *Leucobacter*, which was subsequently recognized as a novel family-level member within the class Actinobacteria.

The genus name *Leucobacter* is derived from the Greek words “leucos” (white) and “bacter” (rod), highlighting the characteristic coloration of certain strains on culture media. Members of this genus are typically Gram-positive, non-spore-forming bacteria distinguished by their unique carotenoid pigments. Notably, they demonstrate a remarkable ability to tolerate high concentrations of metal ions and are well adapted to survive in diverse extreme environments [2].

*C. elegans* is widely recognized as an outstanding model organism for the rapid screening of virulence factors and infection strategies employed by a variety of pathogens. Its small size, ease of cultivation, and well-characterized genetic background make it an ideal system for studying host–pathogen interactions in real time. For example, pathogens such as *Pseudomonas aeruginosa* [3] and *Bacillus cereus* [4] have been shown to cause nematode death through the secretion of toxins or by disrupting critical host signaling pathways. These pathogens utilize a variety of mechanisms to subvert host defenses, and *C. elegans* serves as a powerful tool for identifying and characterizing these virulence factors. 

Despite lacking the complex adaptive immune system in higher organisms, *C. elegans* relies heavily on its innate immune responses to combat infections. This innate immunity is primarily mediated through highly conserved pathways, many of which are shared with mammals. For instance, *C. elegans* modulates the expression of immune-related genes via key signaling pathways such as the p38 MAPK [5] and DAF-2/DAF-16 [6] insulin pathways. These pathways regulate the nematode’s immune response to bacterial infections, cellular stress, and environmental insults. Previous studies have highlighted the importance of these pathways in mediating resistance to bacterial pathogens, providing valuable insights into the evolution of immune responses across species. 

The genus *Leucobacter* was first named in 1996. Despite its formal establishment, research on this genus has remained relatively limited. The earliest described species, *Leucobacter komagatae*, was defined by Takeuchi and Hatano [1]. In 2004, *Leucobacter albus* [7], notable for its resilience in polluted environments, which underscored the genus’s adaptability and biotechnological potential, was characterized by Lin et al. In the same year, Morais et al. named *Leucobacter chromiireducens* and *Leucobacter aridicollis* [8], both of which were isolated from chromium-contaminated environments. The species name *chromiireducens* reflects its remarkable ability to reduce hexavalent chromium (Cr(VI)) to trivalent chromium (Cr(III)), highlighting its potential for heavy metal bioremediation. Subsequently, in 2006, Morais et al. described *Leucobacter luti* and *Leucobacter alluvii* [9], isolated from activated sludge and river sediments, respectively, both of which were exposed to chromium contamination. In 2011, *Leucobacter celer* [10] and *Leucobacter salsicius* [11] were identified by Shin et al. and Yun et al. in food products, while Behrendt et al. and Her et al. discovered *Leucobacter exalbidus* [12], *Leucobacter tardus* [13], and *Leucobacterhumi* [14] in soil environments. These species demonstrate the genus’s adaptability to extreme environmental conditions, including high salinity, low temperatures, and nutrient limitations, and its ability to thrive in dry habitats. Moreover, within the microenvironment of living organisms, *Leucobacter iarius* [15] and *Leucobacter chromiireducens* [16] were isolated from *C. elegans*, suggesting that the *Leucobacter* species may have a specialized ecological affinity for certain environmental niches [17].

*C. elegans* has an exceptionally short lifespan, typically ranging from 2 to 3 weeks under standard laboratory conditions. This short life cycle allows researchers to study multiple generations in a limited time, making it an ideal model for studying the genetic, environmental, and molecular factors that influence aging and longevity [18,19]. The ability to quickly assess the effects of genetic modifications, drugs, or environmental changes on the lifespan of *C. elegans* significantly reduces the time and resources needed compared to studies on longer-lived organisms.

The aging process in *C. elegans* is well characterized and easily observed [20]. As the worms age, they exhibit typical signs of aging, such as reduced mobility, reproductive decline, and increased susceptibility to stress. Due to its short lifespan, *C. elegans* is also an excellent model for investigating the impact of environmental factors (e.g., diet, toxins, and stress) and lifestyle interventions (e.g., caloric restriction or exercise) on aging. 

Despite its simplicity, *C. elegans* shares many conserved molecular pathways with humans, making it an ideal model for studying disease mechanisms [21], drug responses [22], and host–microbe interactions [23]. These similarities further highlight its utility in human health research, particularly in understanding aging and age-related diseases.

The current research on *Leucobacter* species focused on their environmental adaptability, biodegradation abilities, and industrial applications. In contrast, studies exploring their interactions with animal hosts, particularly with the model organism *C. elegans*, are limited. Prior research has primarily examined the interactions between *C. elegans* and other bacterial species, such as *Escherichia coli*, *Pseudomonas aeruginosa*, and members of the *Bacillus* genus. However, the potential effects of *Leucobacter* on *C. elegans* development, reproduction, lifespan, behavior, and overall physiology remain largely unexplored. For example, *C. elegans* is known to respond to bacterial exposure through alterations in immune responses and metabolic states, often mediated by microbial metabolites, toxins, or signaling molecules [24,25,26]. However, whether *Leucobacter* can influence key immune signaling pathways, such as the p38 MAPK and DAF-16/FOXO pathways, or modulate metabolic processes like lipid metabolism and redox balance is largely unknown.

*Leucobacter* may function as a beneficial symbiont that promotes *C. elegans* growth or, conversely, as a conditional pathogen capable of inducing toxicity under specific circumstances. Nevertheless, the mechanisms underlying its interaction with *C. elegans* remain poorly understood and warrant further investigation.

## 2. Results

### 2.1. The Developmental Toxicity of Leucobacter sp. HNU-1 to Caenorhabditis elegans

**Figure 1:**
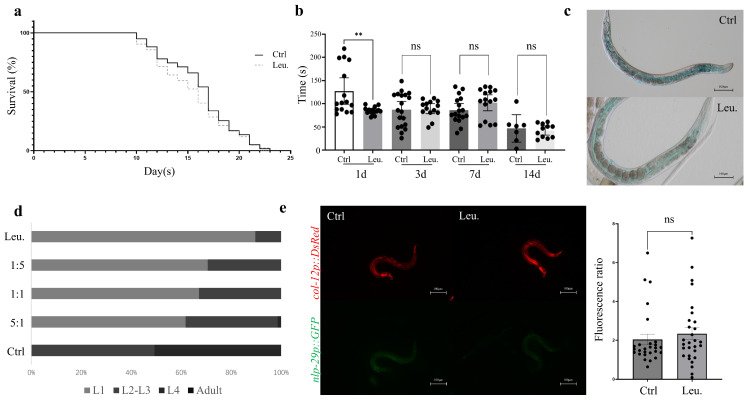
Toxic effect of HNU against *C. elegans*. (**a**) Lifespan of *C. elegans* fed on *E. coli* OP50 (a traditional lab food for *C. elegans*) and *Leucobacter* sp. HNU-1. (**b**) Quantification of pharyngeal pumping rates of *C. elegans* fed on the indicated foods. Pharynx pumping times per 30 s were plotted. (**c**) Intestinal staining with brilliant blue in *C. elegans* with indicated bacterial feeding. (**d**) Developmental defects in *C. elegans* resulting from exposure to HNU-1. *C. elegans* at different developmental stages, i.e., L1, L2, L3, L4 and Adult were quantitated after 48 h of feeding on OP50 (control group), Leu. (experimental group), or varied ratios of mixtures of OP50 and Leu (1:5, 1:1, or 5:1). (**e**) Exposure to HNU-1 does not upregulate nlp-29, an innate immune pathway gene. A representative image of worms fed on OP50 or Leu stably expressing *nlp-29p::GFP* and *col-12p::DsRed*. Quantitative analysis of GFP/DsRed mean fluorescent intensity (MFI) ratios as a measure of the wound response is shown on the right. Error bars represent the Standard Error of the Mean (SEM). Statistical differences were determined using the Student’s *t*-test; **: *p* < 0.01, ns: not significant.

While *C. elegans* shows robust immune responses to a variety of pathogens, the ingestion of HNU-1 does not significantly affect the nematode’s lifespan (*n* = 60, with three biological replicates, Figure 1a) or its locomotor activity (*n* = 15, with three biological replicates, Figure 1b), nor induce detectable intestinal damage (*n* = 20, with three biological replicates, Figure 1c), despite the fact that many infections originate in the gastrointestinal tract [27]. This suggests that HNU-1 may not activate the same harmful effects typically observed with other pathogens that directly damage host tissues. When the environment is unfavorable for growth and development, *C. elegans* may experience immune responses or stress reactions triggered by bacterial metabolites, which could lead to developmental arrest [28]. This phenomenon was observed following ingestion of HNU-1 (*n* = 150, with three biological replicates, Figure 1d), indicating that bacterial toxicity, immune signaling, and host–pathogen interactions may be intricately involved in this process. 

To illuminate these potential interactions between *C. elegans* and HNU-1, *C. elegans* IG274, which contains a cuticular collagen *col-12::DsRed* reporter and the innate immune gene *nlp-29::GFP*, was used to assess immune responses [29]. The epidermal green fluorescence was activated when infected by *Sporangium* or exposure to various stresses, such as epidermal damage or osmotic stress, while DsRed fluorescence serves as a baseline control. The GFP/DsRed ratio is commonly employed as a measure of immune activation or tissue damage in *C. elegans*. However, in this study, ingestion of HNU-1 did not significantly alter the GFP/DsRed ratio (*n* = 30, with three biological replicates, Figure 1e), suggesting that no substantial immune stress response occurred in the nematodes following exposure. This finding further reinforces the notion that *C. elegans* can adapt to environmental challenges in ways that might not invoke the typical immune responses associated with pathogen-induced damage. Moreover, this observation provides valuable insights into the dynamic and sometimes subtle nature of host–pathogen interactions, revealing the complexity of the biological relationships between bacteria and their host organisms. 

### 2.2. Morphological Identification of Leucobacter

**Figure 2:**
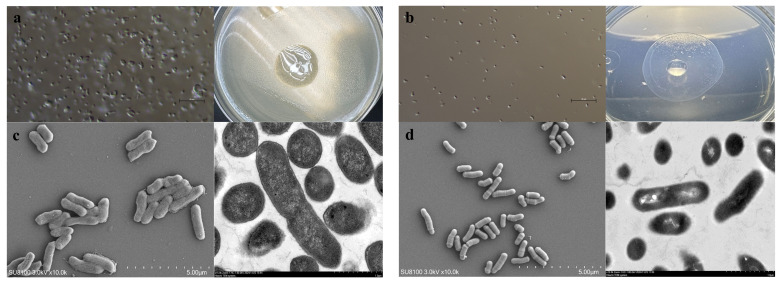
Waterproof property of HNU-1 colony and characterization of its morphology. (**a**,**b**) *E. coli* (**a**) and HNU-1 (**b**) under optical microscope. The *E. coli* colony shows no waterproof property (**a**, right), while the HNU-1 colony is waterproof (**b**, right). (**c**,**d**) Representative images of *E. coli* (**c**) and HNU-1 (**d**) under scanning electron microscope (SEM, left) and transmit electron microscope (TEM, right). (**c**,**d**) The scale bar on the left image (SEM) is 5 µm, while the scale bar on the right image (TEM) is 1 µm.

HNU-1 exhibits a distinct growth pattern, characterized by an initial lag phase lasting from 0 to 9 h, followed by a logarithmic growth phase commencing at 9 h, and culminating in the stationary phase at 25 h (Figure S1). HNU-1 can grow at both 28 °C and 37 °C, with optimal growth observed at 37 °C. Morphologically, HNU-1 is a short, rod-shaped bacterium that does not produce spores. It grows slowly on conventional media, forming smooth, regular colonies with well-defined edges and hydrophobic properties (Figure 2a–d).

Overall, these findings highlight the remarkable adaptability of *C. elegans* in the face of environmental stressors and pathogenic challenges. They also underscore the utility of *C. elegans* as a model organism for studying the molecular and cellular basis of host immune responses, bacterial pathogenesis, and the broader implications of microbial interactions in health and disease.

### 2.3. Biochemical Tests of HNU-1

A comprehensive biochemical characterization of the isolated *Leucobacter* sp. HNU-1 strain was performed to elucidate its metabolic capabilities and physiological properties. Based on the biochemical reaction results (Table 1), HNU-1 exhibited weak cloud-like diffusion in semi-solid agar, indicating limited motility. Additionally, it was unable to utilize citrate, ornithine, inositol, ribitol, or raffinose, suggesting specific constraints in its carbon and nitrogen metabolism pathways.

Regarding amino acid metabolism, HNU-1 tested positive for lysine decarboxylation, indicating lysine decarboxylase activity. However, it failed to hydrolyze urea, implying the absence of urease activity, and did not produce hydrogen sulfide, suggesting it lacks the ability to reduce thiosulfate to H_2_S. In other biochemical assays, HNU-1 tested positive for both the indole and methyl red (MR) tests, demonstrating its capacity to degrade tryptophan into indole and generate stable acidic products through mixed-acid fermentation. In contrast, the Voges–Proskauer (VP) test was negative, indicating that the strain does not synthesize acetoin via the butanediol fermentation pathway.

Furthermore, HNU-1 effectively utilized mannitol, sorbitol, and maltose as carbon sources, suggesting the presence of the necessary carbohydrate-metabolizing enzymes. However, it was unable to deaminate phenylalanine, indicating the absence of phenylalanine deaminase activity. These biochemical characteristics highlight the metabolic specificity of HNU-1, providing fundamental insights for its taxonomic classification and functional studies.

### 2.4. 16S Sequencing, Genome Assembly, and KEGG, GO, and COG Pathway Annotation of HNU-1

Molecular identification was performed using 16S rDNA sequencing. 16S rDNA from HNU-1 was amplified by PCR using genomic DNA extracted from HNU-1 as the template (Figure S2). The purified PCR product, verified through agarose gel electrophoresis, was sequenced to obtain the nucleotide sequence (with three replicates). The resulting 16S rDNA (Figure S3) sequence has been deposited in the GenBank database under accession number PQ664966. A BLASTn search against all available 16S rDNA sequences in the NCBI database showed the highest similarity to the genus *Leucobacter*. Based on morphological, physiological, and biochemical analyses, HNU-1 was preliminarily identified as a member of the genus *Leucobacter*.

The misuse and overuse of antibiotics have emerged as critical global public health challenges in recent years [30]. Antibiotics are introduced into the human body through various pathways, including the food chain, via poultry, livestock, crops, and environmental sources such as water and microorganisms. These practices have significantly accelerated the development of antibiotic resistance, undermining the effectiveness of antimicrobial therapies and facilitating the emergence of superbugs and resistant viruses. Genomic sequencing of multidrug-resistant microorganisms is pivotal for elucidating the underlying mechanisms of resistance and developing targeted strategies to combat these pathogens. Addressing these issues is essential to curbing antibiotic misuse. 

**Figure 3:**
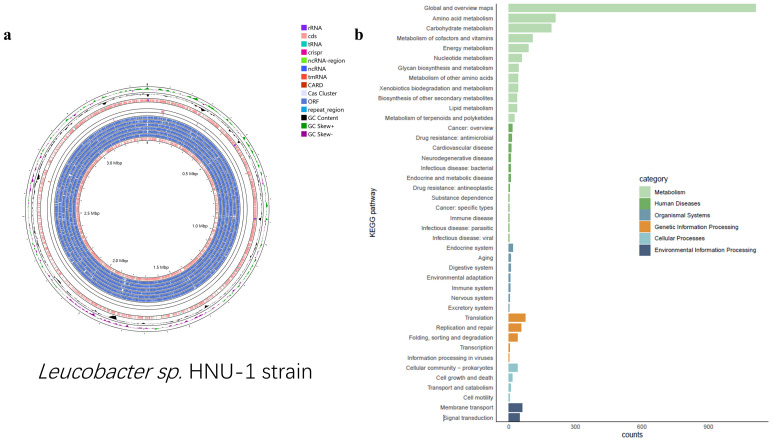
Nanopore sequencing and assembly of HNU-1 genome. (**a**) Chromosomal genomic map of *Leucobacter* sp. HNU-1. Starting from the outermost ring: Ring 1: GC skew (the specific formula is G − C/G + C, which measures the relative content of G and C. If G > C, the GC skew value is positive and is represented by the inward pink region; if G < C, it is negative and represented by the outward light green region.). Ring 2: GC content (the inward red region indicates that the GC content in this area is lower than the average GC content of the entire genome, while the outward green region indicates the opposite. The higher the peak, the greater the difference from the average GC content). Ring 3: Prokka annotation (+). Ring 4: CARD RGI results (+). Ring 5: Backbone (*Leucobacter* sp. HNU-1). Ring 6: CARD RGI results (−). Ring 7: CRISPRCasFinder annotation (+). Ring 8: CRISPRCasFinder annotation (−). Ring 9: ORFs (+3). Ring 10: ORFs (+2). Ring 11: ORFs (+1). Ring 12: ORFs (−1). Ring 13: ORFs (−2). Ring 14: ORFs (−3). Ring 15: Prokka annotation (−). (**b**) Details of KEGG analysis of *Leucobacter* sp. HNU-1. There are six categories, as shown on the right side of Figure 2b: each category is divided into a secondary classification system: *x*-axis represents the number of genes, and the *y*-axis represents the biological pathway. In secondary classification, different colors are used to distinguish the primary classification of biological pathways.

**Figure 4:**
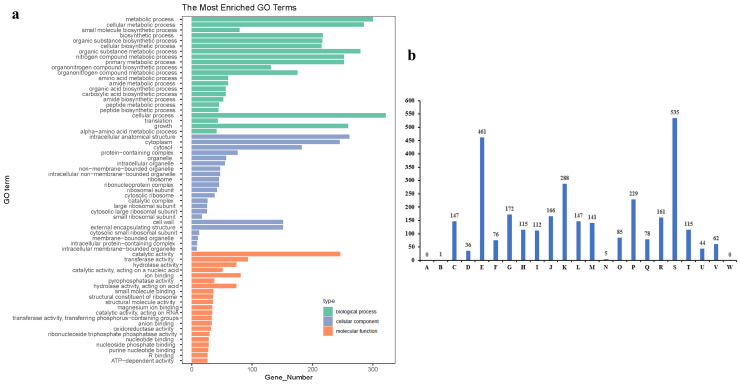
Gene enrichment analysis of the HNU-1 genome. (**a**) GO functional classification of *Leucobacter* sp. HNU-1. The *x*-axis represents the number of genes, the *y*-axis represents the GO term, and different colors are used to distinguish biological processes, cell components, and molecular functions. (**b**) COG functional annotation classification of *Leucobacter* sp. HNU-1 A: RNA processing and modification; B: energy production and conversion; C: cell cycle control, cell division, and chromosome partitioning; D: amino acid transport and metabolism; E: nucleotide transport and metabolism; F: carbohydrate transport and metabolism; G: coenzyme transport and metabolism; H: lipid transport and metabolism; I: translation, ribosomal structure, and biogenesis; J: transcription; K: replication, recombination, and repair; L: cell wall/membrane/envelope biogenesis; M: cell motility; N: posttranslational modification, protein turnover, and chaperones; O: inorganic ion transport and metabolism; P: secondary metabolite biosynthesis, transport, and catabolism; Q: general function prediction only; R: function unknown; S: signal transduction mechanisms; T: intracellular trafficking, secretion, and vesicular transport; U: defense mechanisms; V: extracellular structures; W: mobilome: prophages and transposons.

In this context, whole-genome sequencing of the *Leucobacter* sp. HNU-1 was undertaken to provide comprehensive insights into its multidrug resistance and to support future research aimed at identifying potential therapeutic interventions. The genomic analysis revealed a genome length of 3,375,033 bp, with an average coding gene length of 15,672 bp, a GC content of 70.37%, and a total of 3270 protein-coding genes, 9 rRNA genes, and 52 tRNA genes (Figure 3a). Functional annotation of all genes was performed using the Kyoto Encyclopedia of Genes and Genomes (KEGG), Gene Ontology (GO), and Clusters of Orthologous Groups (COGs) databases. The KEGG database classifies biological metabolic pathways into six categories [31]. In HNU-1, 2492 annotated genes, representing 5.01% of the total coding genes, were associated with various functions and metabolic pathways. Of these, 80.30% are involved in metabolism, 7.02% in genetic information processing, 4.37% in cellular processes, 2.73% in environmental information processing, 2.24% in human diseases, and 3.33% in organismal systems. Each of these categories contains specific subcategories, allowing for the identification of genes involved in the regulation—upregulation or downregulation—of target metabolites (Figure 3b). In the GO database, genomic information is classified into three main categories: molecular function (M), biological process (P), and cellular component (C) [32]. In *Leucobacter* sp. HNU-1, 2082 genes were annotated in the GO database, accounting for 63.67% of the total coding genes, with a predominant involvement in molecular functions. The statistical results are shown in Figure 4a. The COG database was used to classify phylogenetic relationships between proteins encoded by the complete genome of HNU-1 [33]. A total of 2867 annotated genes, or 87.68% of the total genes, were categorized. Among these, 18.66% (535 genes) are associated with signal transduction mechanisms (S), 16.08% to nucleotide transport and metabolism (E), 10.04% (288 genes) with replication, recombination, and repair (K), and 7.99% (229 genes) with the biosynthesis, transport, and catabolism of secondary metabolites (P). Additionally, 2.72% of genes have general functional predictions, and 5.62% have unknown functions, indicating the potential for further functional research (Figure 4b).

### 2.5. Carbohydrate-Active Enzymes Pathway Annotation of HNU-1

**Figure 5:**
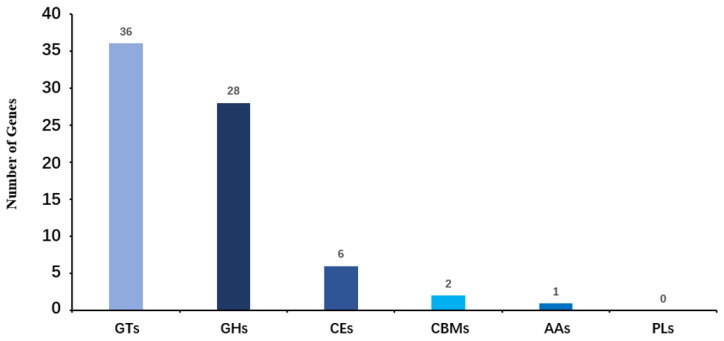
Functional annotation and classification of carbohydrate-active enzymes (CAZy) in the genome of *Leucobacter* sp. HNU-1. The horizontal axis represents the classification of enzymes (different colors represent different types of enzymes), and the vertical axis represents the number of genes contained in these classifications.

The functional annotation of *Leucobacter* sp. HNU-1 genes in the CAZy (Carbohydrate-Active Enzymes [34]) database is summarized in Figure 5. A total of 73 genes encoding carbohydrate-active enzymes were identified, reflecting the bacterium’s potential involvement in diverse carbohydrate metabolism processes. Among these, 28 genes were classified as glycoside hydrolases (GHs), which are responsible for breaking glycosidic bonds in complex carbohydrates, facilitating their degradation and utilization. Another 36 genes were annotated as glycosyltransferases (GTs), enzymes essential for the synthesis of glycosidic bonds, suggesting an active role in the biosynthesis of polysaccharides and glycoconjugates. 

In addition, two genes were identified as encoding carbohydrate-binding modules (CBMs), which are often associated with other enzyme domains and play a critical role in recognizing and binding specific carbohydrate substrates. Six genes were assigned to the carbohydrate esterase (CEs) family, enzymes that act on ester linkages within carbohydrates, contributing to their structural modification. One gene was annotated as an auxiliary activity enzyme (AAs), which typically supports oxidative breakdown of polysaccharides and other carbohydrate polymers.

Interestingly, no genes from the polysaccharide lyase (PLs) family were detected in the *Leucobacter* sp. HNU-1 genome, indicating a deficiency in enzymatic machinery for cleaving polysaccharides through a β-elimination mechanism in *Leucobacter* sp. HNU-1. This absence may suggest a unique adaptation or a limited role in specific polysaccharide degradation pathways compared with other bacteria. Overall, these findings highlight the diverse enzymatic toolkit of HNU-1 for carbohydrate metabolism, providing insights into its ecological functions and metabolic capabilities.

### 2.6. CARD Antibiotic Resistance Prediction and K-B Antimicrobial Susceptibility Testing of HNU-1

In addition, a comprehensive analysis using the Comprehensive Antibiotic Research Database (CARD) [35] revealed five antibiotic-resistant genes and secondary metabolite bio-synthetic gene clusters within the HNU-1 genome. The identified clusters predominantly include two vancomycin resistance genes: the vanY homolog in the vanB cluster and another vanY homolog in the vanG cluster, both of which are implicated in resistance to this critical glycopeptide antibiotic. Furthermore, a sulfonamide resistance gene (su11) and an aminoglycoside resistance gene (aadA2) were also identified, contributing to HNU-1’s resistance to these commonly used antibiotics (Table 2). These findings suggest that HNU-1 harbors multiple resistance mechanisms, which could influence its response to antibiotic treatments.

Resistance genes such as *bla*, *sul*, and *van* confer resistance to β-lactam, sulfonamide, and vancomycin antibiotics, respectively. The *bla* gene encodes β-lactamase, an enzyme that hydrolyzes the β-lactam ring, thereby inactivating the antibiotic. The *sul* gene encodes a variant enzyme that exhibits reduced sensitivity to sulfonamide inhibition. In contrast, the *van* gene alters the vancomycin target site, decreasing the drug’s binding affinity. Collectively, these mechanisms significantly enhance bacterial resistance to multiple classes of antibiotics.

In this study, we evaluated the antibiotic susceptibility of an isolated strain using 30 commonly employed antimicrobial agents and the Kirby–Bauer disc diffusion method, in accordance with Clinical and Laboratory Standards Institute (CLSI) guidelines. The results, as shown in Table 3, revealed that the strain exhibited marked resistance to several classes of antibiotics, including β-lactams (oxacillin [OX], ceftriaxone [CTR], cefoperazone [CPZ]), aminoglycosides (kanamycin [KAN], streptomycin [S]), polymyxin B (PB), lincosamides (clindamycin [CC], lincomycin [MY]), carbapenems (imipenem [IPM]), fluoroquinolones (ciprofloxacin [CIP], norfloxacin [NOR]), and sulfonamides (trimethoprim-sulfamethoxazole [SXT]). Additionally, the strain exhibited intermediate resistance to vancomycin, erythromycin, and tetracycline. Conversely, it was sensitive to selected antibiotics within the same classes, including β-lactams (penicillin, ampicillin, cefuroxime sodium, cefalexin, piperacillin, ceftazidime), tetracyclines (minocycline, doxycycline), chloramphenicol derivatives (chloramphenicol, florfenicol), fluoroquinolones (levofloxacin), aminoglycosides (gentamicin, amikacin), and macrolides (azithromycin). The underlying resistance mechanisms warrant further investigation.

The increasing prevalence of multidrug-resistant bacteria poses significant challenges for clinical treatment. Resistance is mediated by a suite of virulence genes that enhance bacterial persistence and pathogenicity, promoting both colonization and tissue invasion. These findings underscore the broad-spectrum resistance of the HNU-1 strain and its potential implications for clinical infection control and environmental microbiology.

### 2.7. Construction of the Phylogenetic Tree of HNU-1

**Figure 6:**
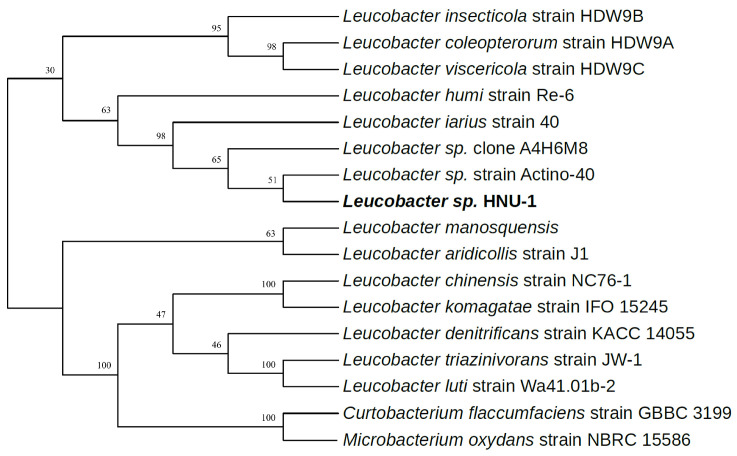
Phylogenetic placement of HNU-1 among related bacteria. The phylogenetic tree was reconstructed based on the 16S rDNA genomes of 15 strains belonging to the genus *Leucobacter*. *Curtobacterium flaccumfaciens* strain GBBC 3199 and *Microbacterium oxydans* strain NBRC 15586 were designated as outgroups.

A phylogenetic tree of seven *Leucobacter* genomes was reconstructed using the Construct/Test Neighbor-Joining Tree method [36,37]. *Curtobacterium flaccumfaciens* strain GBBC 3199 (CP041259.1, 78.19% similarity) from the family *Microbacteriaceae,* genus *Curtobacterium* and *Microbacterium oxydans* strain NBRC 15586 (CP162522.1, 77.35% similarity) from the family *Microbacteriaceae*, genus *Microbacterium* were used as outgroups. Phylogenetic analysis revealed that 15 *Leucobacter* species formed two distinct clades. Among them, five species—*Leucobacter triazinivorans*, *Leucobacter luti*, *Leucobacter denitrificans*, *Leucobacter chinensis*, and *Leucobacter komagatae*—grouped into a terminal branch, whereas the remaining eight species formed a monophyletic cluster nested within the evolutionary lineage of *Leucobacter* sp. (Figure 6). Notably, *Leucobacter* sp. HNU-1 was closely related to *Leucobacter iarius*.

### 2.8. Comparative Genomics of HNU-1 and Six Leucobacter Species, and ANI Analysis of All Leucobacter Genomes Available on NCBI

To further explore genomic relationships, the genome of *Leucobacter* sp. HNU-1 was compared with six other reference genomes within the genus available in the NCBI database. Among these genomes, *Leucobacter iarius* and *Leucobacter* sp. HNU-1 shared similar chromosome lengths, numbers of coding sequences (CDSs), and tRNA counts, all of which were higher than those of three other strains. Their GC content also showed consistent patterns. The number of tRNAs was comparable across the seven strains, averaging around 50 per genome. Notably, the *Leucobacter* sp. HNU-1 genome contained six CRISPR structures, whereas *Leucobacter iarius* JCM 14736 harbored 13, and the other strains typically contained 1–3 (Table 4). Average Nucleotide Identity (ANI) [38] was calculated to assess genetic relatedness at the nucleotide level by comparing homologous genomic segments, as described by Goris et al. in 2007 [39]. ANI values above 95% generally indicate conspecific genomes, while values below 75% are considered unreliable. ANI analysis of *Leucobacter* sp. HNU-1 against 149 known *Leucobacter* genomes in the NCBI database revealed that all strains had ANI values exceeding 75%. However, only one strain, GCA_040392405.1, exceeded the 95% threshold, with an ANI of 96.76%. This value was deemed unreliable due to the small genome size (2.2 Mb) of GCA_040392405.1. Consistent with these findings, ANI values below the interspecies threshold of 95% and the independent evolutionary branch observed in the 16S rDNA phylogenetic tree support the classification of *Leucobacter* sp. HNU-1 as a distinct variant within the *Leucobacter* genus (Table S1). 

To further support species delineation, digital DNA–DNA hybridization (dDDH) analysis was performed between strain HNU-1 and *Leucobacter iarius* JCM 14736, yielding a value of 43.8%. This value is significantly below the 70% threshold commonly used to define prokaryotic species boundaries [40], indicating that HNU-1 represents a distinct genomic lineage. In combination with its unique phenotypic traits, most notably, its ability to induce non-dauer developmental arrest in *C. elegans*, these findings provide strong evidence for the classification of strain HNU-1 as a novel species within the genus Leucobacter (Supplementary Table S2).

A combination of phylogenetic and genome-based analyses, including 16S rRNA similarity, ANI, and dDDH, along with distinct phenotypic traits, supports the classification of strain HNU-1 as a novel species within the genus Leucobacter.

Based on the results of phylogenetic tree analysis, six *Leucobacter* reference genomes *Leucobacter iarius* JCM 14736, *Leucobacter chromiireducens* TAN 31504, *Leucobacter aridicollis* DSM 17380, *Leucobacter coleopterorum* HDW9A, *Leucobacter komagatae* DSM 8803, and *Leucobacter luti* RF6 were selected for synteny analysis with the *Leucobacter* sp. HNU-1 genome using Mauve. This approach allowed for the comparison of gene order and structural variation across the genomes, shedding light on their evolutionary relationships. 

### 2.9. Bacterial Synteny Analysis of HNU-1 and Six Leucobacter Species

**Figure 7:**
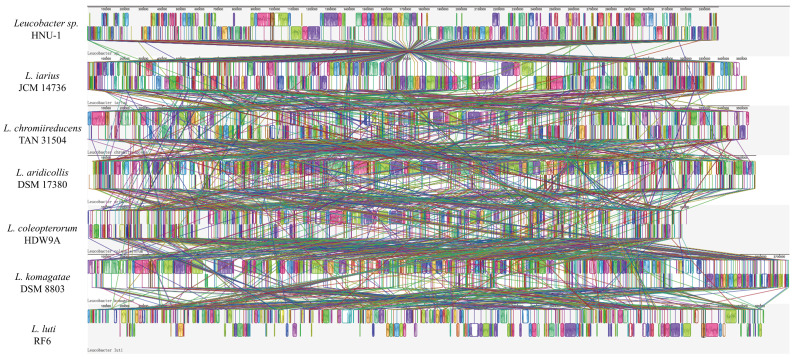
Genome synteny analysis of *Leucobacter* sp. HNU-1 and related strains. The synteny of *Leucobacter* sp. HNU-1 was analyzed in comparison with *L. iarius* JCM 14736, *L. chromiireducens* TAN 31504, *L. aridicollis* DSM 17380, *L. coleopterorum* HDW9A, *L. komagatae* DSM 8803, and *L. luti* RF6. Colored regions in the visualization represent conserved syntenic segments across the genomes.

As depicted in Figure 7, *Leucobacter* sp. HNU-1 demonstrated strong synteny with *L. iarius* JCM 14736, with substantial conservation of gene order across large genomic regions. However, notable genomic rearrangements, including insertions, deletions, inversions, and translocations, were observed, highlighting significant evolutionary divergence between the two strains. 

In contrast, the synteny analysis of *Leucobacter* sp. HNU-1 with the other five reference genomes (*L. chromiireducens* TAN 31504, *L. aridicollis* DSM 17380, *L. coleopterorum* HDW9A, *L. komagatae* DSM 8803, and *L. luti* RF6) revealed considerable differences in the arrangement of Locally Collinear Blocks (LCBs) [41]. These variations in LCBs suggest that while the *Leucobacter* species share a common evolutionary origin, their genomes have diverged significantly over time, likely reflecting adaptations to different ecological niches or functional specializations (Figure 7). The analysis underscores the complex genomic architecture within the *Leucobacter* genus and provides insights into the genomic flexibility that may contribute to the diversity of metabolic and ecological functions observed in this group of bacteria. 

## 3. Discussion

In this study, *Leucobacter* sp. HNU-1 was identified as a bacterium capable of inducing developmental delay in *C. elegans* without causing significant effects on the nematode’s lifespan, motility, or intestinal morphology. Dauer formation is a specialized developmental state in *C. elegans* that typically occurs under unfavorable environmental conditions, such as high population density [42] or limited food availability [43]. 

Interestingly, we found that the developmental arrest induced by *Leucobacter* sp. HNU-1 was not a dauer state. Specifically, under our experimental conditions, HNU-1 had no effect on the lifespan of *C. elegans*. Moreover, a standard assay for dauer confirmation involves treatment with 1% sodium dodecyl sulfate (SDS), to which dauer larvae are resistant due to the closure of their mouth and anus, preventing SDS penetration. In contrast, all worms arrested by HNU-1 exposure were killed by SDS treatment, indicating that they underwent a general developmental delay rather than entering dauer.

The ability of HNU-1 to induce this arrest in the absence of environmental stressors suggests a novel mode of host–microbe interaction. Notably, this effect differs from previously described trauma-like responses, as ingestion of HNU-1 did not lead to increased *nlp-29*::GFP fluorescence, a marker of immune activation in *C. elegans*. These results suggest that HNU-1 does not activate canonical immune response pathways typically triggered by pathogenic bacteria.

Further analysis using electron microscopy revealed that HNU-1 is morphologically distinct from the widely used laboratory food source *Escherichia coli* OP50. Specifically, HNU-1 cells are smaller in both length and width, lack prominent membrane structures, and exhibit a hydrophobic surface. These physical characteristics may influence the interaction between HNU-1 and the intestinal environment of *C. elegans*. Whole-genome sequencing provided additional insights into the unique features of HNU-1, uncovering a genome enriched with genes associated with molecular synthesis processes, signal transduction pathways, and nucleotide transport and metabolism. These genomic features suggest that HNU-1 has a complex metabolic capacity, which may play a role in its ability to induce developmental delay in *C. elegans*. Such interactions between bacterial metabolites and host signaling pathways may represent a novel mechanism underlying the developmental delay phenomenon, warranting further molecular investigation.

In addition to its developmental delay properties, HNU-1 was found to harbor multiple antibiotic resistance genes through analysis of the Comprehensive Antibiotic Resistance Database (CARD). The presence of these resistance genes raises intriguing questions about the potential role of bacterial antibiotic metabolites in modulating host developmental pathways. While the specific contribution of these resistance-related genes to developmental delay remains unclear, future studies could explore whether bacterial antibiotic production affects the nematode’s physiology or behavior.

Comparative genomic analyses highlighted the distinctiveness of HNU-1 when compared to other *Leucobacter* species with complete genomes available in the NCBI database. Phylogenetic analysis based on the 16S rDNA sequences showed that HNU-1 forms a relatively independent branch within the *Leucobacter* genus, further supporting its classification as a novel variant species. These findings suggest that HNU-1 is evolutionarily unique and distinct from previously characterized *Leucobacter* species. The discovery expands understanding of the diversity within the *Leucobacter* genus and underscores the importance of exploring bacterial species in under-studied environments.

To the best of our knowledge, previously identified *Leucobacter* strains, such as *Leucobacter komagatae* (first isolated in 1996), have not been reported to exhibit resistance to as many antibiotics as *Leucobacter* sp. HNU-1. Furthermore, these strains have not been studied in the context of host-microbe interactions. HNU-1 was originally isolated from a crocodile farm experiencing severe disease outbreaks. Notably, we found that this strain impairs the development of *C. elegans*, suggesting that its virulence may be evolutionarily conserved. The combination of pathogenicity and extensive antibiotic resistance distinguishes HNU-1 as a unique *Leucobacter* model, underscoring its relevance for further research.

The antibiotic resistance genes identified in *Leucobacter* sp. HNU-1 are likely encoded in its genome rather than on plasmids. Despite this, HNU-1 may still pose ecological and human health risks as an environmental reservoir of antibiotic resistance genes, which could be horizontally transferred to other bacteria as large genomic DNA fragments or via bacteriophages.

The comprehensive genomic and phenotypic analysis of HNU-1 provides a theoretical foundation for further research on antibiotic-resistant bacteria in tropical regions of China. As a bacterium with both unique genomic features and the ability to modulate host development, HNU-1 offers a valuable model for studying host–bacterial interactions and their implications for microbial ecology, evolutionary biology, and medical research. These findings contribute to the growing body of knowledge on the role of environmental bacteria in influencing the behavior and physiology of host organisms.

## 4. Materials and Methods

### 4.1. Isolation of HNU-1 and Extraction of Genomic DNA

HNU-1 was isolated from wastewater in the tropical environment of Hainan, China, using the method of Her et al. [14] for extracting *Leucobacter humi* from soil: 1 mL of sewage was inoculated into 100 mL of 1/10 diluted nutrient broth and incubated aerobically in a shaking incubator (150 rpm, 28 °C) for 3 days. After enrichment, 100 µL of the sample was spread onto a 1/10 diluted nutrient agar (LA) plate and incubated at 28 °C for 2 days. Single colonies were then inoculated into 100 mL of liquid LB medium (containing 1 g of tryptone, 1 g of sodium chloride, and 0.5 g of yeast extract) and cultured at 180 rpm and 37 °C for 48 h. One portion of the bacterial culture was stored at −80 °C with 50% glycerol (*v/v*) for future use, while another portion was used for genomic DNA extraction following. We extracted the total genomic DNA of Leucobacter sp. HNU-1 using the Rapid Bacterial Genomic DNA Isolation Kit (Shenggong Biological Co., Ltd., Shanghai, China).

The quality and concentration of the genomic DNA were assessed via agarose gel electrophoresis and NanoDrop analysis. All culture media and solutions were sterilized at 121 °C for 20 min. The *Leucobacter* sp. HNU-1 is currently preserved at Hainan University.

### 4.2. Characterization and Molecular Identification of Leucobacter sp. HNU-1

The identification of *Leucobacter* sp. HNU-1 was based on a combination of morphological, physiological, biochemical, and molecular characteristics. Morphological examination was performed using a Nikon Ni-C differential interference fluorescence microscope (Nikon Corporation, Tokyo, Japan). Hitachi Regulus 8100 scanning electron microscope (Hitachi, Ltd., Tokyo, Japan). And Hitachi HT7800 transmission electron microscope (Hitachi, Ltd., Tokyo, Japan). The hydrophobicity of *Leucobacter* strain HNU-1 was initially observed during our *C. elegans* experiments. We prepared NGM plates, onto which overnight-cultured HNU-1, or OP50 were spotted. The plates were then incubated at 37 °C overnight. When H_2_O was introduced onto the bacterial lawn, we observed a distinct hydrophobic interaction between H_2_O and the bacterial surface. For molecular identification, the 16S rDNA gene was amplified using the universal bacterial primers 27F (AGAGTTTGATCCTGGCTCAG) and 1492R (GGTTACCTTGTTACGACTT). The amplified fragment was sequenced, and the obtained sequence was compared with known sequences in GenBank using nucleotide BLAST to identify the bacterial species. Based on the results of these analyses, the strain was classified as *Leucobacter* sp. HNU-1.

### 4.3. C. elegans Strains and Maintenance

The *C. elegans* N2 strain was maintained on nematode growth medium (NGM) plates seeded with bacteria (*E. coli* OP50) at 20 °C.

The following strains/alleles were obtained from the Caenorhabditis Genetics Center (CGC) or as indicated:

N2 Bristol (wild-type control strain);

IG274: frIs7 [*nlp-29p::GFP* + *col-12p::DsRed*] IV.

### 4.4. Preparation of Leucobacter sp. (HNU-1) and OP50 Mixed Suspension

To prepare *Leucobacter* sp. (HNU-1), an overnight culture in LB broth (grown at 37 °C) was diluted 1:100 in fresh LB broth. Once the culture reached an optical density of OD600 = 0.5, *Leucobacter* sp. was spread evenly on NGM plates.

For the preparation of mixed bacterial suspensions, 50 μL of *Escherichia coli* OP50 and 50 μL of *Leucobacter* sp. were combined at ratios of 1:5, 1:1, and 5:1. A total of 100 μL of the resulting mixture was then spread uniformly onto NGM plates.

Approximately 100–200 synchronized L1-stage *C. elegans* were transferred onto the designated plates (containing either *Leucobacter* sp. alone or a mixture of *E. coli* OP50 and *Leucobacter* sp.) and incubated at 20 °C to observe and analyze growth phenotypes.

### 4.5. Observation of C. elegans Lifespan

Synchronized *C. elegans* were exposed to NGM medium for 72 h until they reached the L4 stage, after which they were transferred to NGM medium containing 5-fluorouracil. A total of 20 *C. elegans* were transferred to each plate. The number of deceased worms was recorded every 24 h until all individuals had died, and the experiment was repeated three times. A picker was used to gently touch the tails and heads of the worms; individuals that did not respond were considered dead [44]. The young adult stage of *C. elegans* was designated as the initial exposure stage, during which the worms were exposed to the treated NGM medium. Each experimental group included three biological replicates, with 60 *C. elegans* per parallel experiment. Experimental data were visualized using GraphPad Prism 9.5.0 software.

### 4.6. Analysis of Fluorescence Intensity in Worms

For fluorescence imaging (*nlp-29p::GFP* + *col-12p::DsRed*), the worms were anesthetized with 25 mM levamisole, and images were captured using a Nikon Ni-C upright differential interference contrast fluorescence microscope equipped with a CMOS camera (Nikon Corporation, Japan). The GFP/DsRed ratio was calculated to quantify fluorescence intensity. The entire worm region was outlined and quantified using ImageJ 1.38e/Java 1.5.0_09 software, and the fluorescence intensity was normalized for comparison.

### 4.7. Complete Genome Sequencing and Annotation

The draft genome of HNU-1 was sequenced and annotated by BGI Technology Co., Ltd. (Wuhan, China) using the DNBSEQ [45] and Nanopore [46] platforms. Sequencing was performed on the DNBSEQ-T10×4RS (BGI Group, Shenzhen, China) and PromethION instruments (Oxford Nanopore Technologies, Oxford, UK). To ensure the accuracy of the data, raw reads were processed to remove low-quality sequences, adapters, and short reads using the default parameters of the Porechop 0.2.4 software. Nanopore reads were assembled with Flye [47,48]. Genome polishing was carried out using Pilon 1.22 [49] with the Quiver algorithm from the Genomic Consensus package. Gene prediction was performed using Prodigal 2.6.3 [50] with default settings. Transfer RNAs (tRNAs) were identified using the tRNAscan-SE [51] program, and ribosomal RNAs (rRNAs) were annotated using RNAmmer [52]. Gene annotation was performed using Diamond 0.8.15 against the database. Functional annotations of the predicted genes were obtained using Blast2Go 2.5, using the Gene Ontology (GO) database, while pathway annotations were assigned via Blast 2.2.28+ with the KEGG database. Phylogenetic classification of protein-coding genes was carried out using Hmmscan 3.1b2 from the COG database. Enzymes involved in carbohydrate degradation, synthesis, and modification were annotated with Diamond 0.8.15 (E-value 0.00001) to provide catalytic structural and functional details from the CAZy database. Finally, Circos 0.69 software [53] was used to generate a circular visualization of coding sequences (CDSs), non-coding RNAs (ncRNAs), GC content, repetitive sequences, and rRNA.

### 4.8. 16S rDNA Phylogenetic Tree, ANI Analysis, Comparative Genomics, and Synteny Analysis

The phylogenetic relationship between *Leucobacter* sp. HNU-1 and other Leucobacter species was determined based on their 16S rDNA sequence. Using BLASTn, 15 complete 16S rDNA sequences were retrieved from the NCBI nucleotide database for phylogenetic analysis. The strains *Curtobacterium flaccumfaciens* GBBC3199 (CP041259.1, 78.19% similarity) from the family *Microbacteriaceae*, genus *Curtobacterium*, and *Microbacterium oxydans* NBRC15586 (CP162522.1, 77.35% similarity) from the family *Microbacteriaceae*, genus *Microbacterium*, were used as outgroups. Phylogenetic trees were constructed using the Neighbor-Joining method [36] in MEGA11, employing the p-distance model and performing Bootstrap analysis with 1000 replications to assess the robustness of the tree.

Average Nucleotide Identity (ANI) [39] analysis was conducted to evaluate genomic relatedness between *Leucobacter* sp. HNU-1 and other strains. Using the MUMmer alignment method, average ANI values were calculated for 33 genomes. Genome accession numbers for numerous *Bacillus* species are available in GenBank.

Based on the phylogenetic distances inferred from the 16S rDNA tree, complete genome sequences for all *Leucobacter* species were downloaded from the NCBI database for comparative genomics analysis. In this analysis, both ANI and dDDH (digital DNA-DNA hybridization) values were used to assess genomic similarity. Strains with an ANI greater than 95% or a dDDH value greater than 70% are considered to belong to the same species [54,55]. ANI values for *Leucobacter* sp. HNU-1 were calculated using the Kostas Lab ANI Calculator [56] (http://enve-omics.ce.gatech.edu/ani/index, accessed on 4 December 2024) and compared with reference strains *Leucobacter iarius* JCM 14736, *Leucobacter chromiireducens* TAN 31504, *Leucobacter aridicollis* DSM 17380, *Leucobacter coleopterorum* HDW9A, *Leucobacter komagatae* DSM 8803, and *Leucobacter luti* RF6. Genomic structure and synteny between *Leucobacter* sp. HNU-1 and the reference strains were analyzed using the Mauve program within Geneious R9 software (Align with progressiveMauve) [41].

### 4.9. Biochemical Identification of the Isolated Strain Using 16 Biochemical Tests

A total of 16 biochemical characteristics were selected for the biochemical identification of the isolated strain, including the following tests: semi-solid agar motility, ornithine decarboxylase, lysine decarboxylase, citrate utilization, H_2_S production, urease activity, indole test, methyl red (MR) test, Voges-Proskauer (VP) test, phenylalanine deamination, mannitol utilization, inositol utilization, sorbitol utilization, maltose utilization, ribitol utilization and raffinose utilization. For the identification procedure, a single colony of HNU-1, initially isolated on a plate, was picked and suspended in a 0.9% saline solution to achieve the appropriate turbidity. Biochemical tests were then carried out following the guidelines provided by Qingdao Hi-tech Industrial Park Hope Bio-technology Co., Ltd., Qingdao, China.

### 4.10. Prediction of Antibiotic Synthesis Gene Clusters and Analysis of Associated Genes

The Comprehensive Antibiotic Research Database (CARD) integrates antibiotic resistance ontology (ARO) with curated sequences of antimicrobial resistance (AMR) genes (ARGs) and resistance-conferring mutations, providing an informatics framework for the annotation and interpretation of resistance gene clusters. Gene cluster prediction for *Leucobacter* sp. HNU-1 was performed using the CARD database, which includes both strict and perfect hits, as well as those that are nudged from loose to strict hits. Short contigs were excluded from partial gene predictions. Gene sequences with ≥85% similarity to known gene cluster sequences were classified as encoding compounds already identified, while sequences with <85% similarity were considered to encode potentially novel compounds [35,57].

### 4.11. Antibiotic Susceptibility Testing Using the K-B Disk Diffusion

Antibiotic susceptibility testing was conducted using the Kirby-Bauer disk diffusion method, following the guidelines recommended by the Clinical and Laboratory Standards Institute (CLSI). In a biosafety cabinet, 100 µL of a prepared HNU-1 suspension was evenly spread onto an LB agar plate using a sterile swab. After the inoculum dried, sterile forceps were used to place the antibiotic discs gently onto the inoculated agar surface (care was taken to avoid puncturing the plate). The plates were then incubated at 37 °C for 16 h. The diameter of the zone of inhibition was measured with a ruler, and the susceptibility of the HNU-1 strain to each antibiotic was determined based on the criteria provided in the antibiotic disk instructions. Three replicates were performed for each antibiotic. The following 30 antibiotics were tested: penicillin, vancomycin, oxacillin, levofloxacin, clindamycin, erythromycin, polymyxin B, gentamicin, lincomycin, minocycline, tetracycline, chloramphenicol, imipenem, doxycycline, azithromycin, ceftriaxone, ceftazidime, cefoperazone, ciprofloxacin, norfloxacin, florfenicol, piperacillin, streptomycin, compound sulfonamides, ampicillin, kanamycin, amikacin, cefuroxime, cephalexin, and cefamezin. Results were reported based on the criteria of resistant, intermediate, or susceptible.

### 4.12. Complete Nucleotide Sequence and Strain Accession Numbers

The complete nucleotide sequence of *Leucobacter* sp. HNU-1 has been deposited in the GenBank database under the accession number PRJNA1137138. The strain is available from the Environmental Health and Public Health Research Laboratory, Hainan University, Haikou, Hainan Province, China, with postal code 570228.

## 5. Conclusions

*Leucobacter* sp. HNU-1 significantly affects the morphological and physiological characteristics of *Caenorhabditis elegans*, particularly in inducing developmental delay. Although HNU-1 does not notably impact the nematode’s lifespan, motility, or intestinal morphology, it induces a developmental delay in the absence of external environmental stress, revealing a novel interaction between the bacterium and its host. Furthermore, the genomic analysis of HNU-1 reveals a complex metabolic capacity, particularly genes associated with molecular synthesis, signal transduction, and nucleotide metabolism, which may contribute to the induction of the developmental delay phenotype. HNU-1 also harbors multiple antibiotic resistance genes, suggesting its potential role in complex host–bacterial interactions. Overall, this study identifies *Leucobacter* sp. HNU-1 as a novel species within the *Leucobacter* genus, offering new insights into host–bacterial interactions and providing a novel research direction for exploring non-traditional immune pathways and cellular stress responses triggered by bacteria. These findings have significant biological and medical implications.

## Figures and Tables

**Table 1:** Biochemical test results for the isolated *Leucobacter* sp. HNU-1 strain.

Biochemical Test	Result
Semi-solid agar motility	+
Ornithine decarboxylase	−
Lysine decarboxylase	+
Citrate utilization	−
H_2_S production	−
Urease	−
Indole test	+
Methyl red (MR) test	+
Voges–Proskauer (VP) test	−
Phenylalanine deamination	−
Mannitol utilization	+
Inositol utilization	−
Sorbitol utilization	+
Maltose utilization	+
Ribitol utilization	−
Raffinose utilization	−

**Note:** “+” indicates a positive result; “−” indicates a negative result.

**Table 2:** Antibiotic resistance gene analysis of *Leucobacter* sp. HNU-1.

Antibiotic	Start	Stop	AMR Gene Family	Best Identities	Orientation
vancomycin	418,532	419,158	vanY;glycopeptide resistance gene cluster	31.71	+
vancomycin	659,487	660,260	vanY;glycopeptide resistance gene cluster	38.93	+
sulfadiazine	2,675,593	2,676,432	sulfonamide resistant sul	100	−
spectinomycin	2,676,937	2,677,716	ANT(3″)	100	−
defensin	1,174,301	1,174,543	defensin resistant mprF	100	−

**Table 3:** Antimicrobial susceptibility testing results. According to the guidelines provided by the Clinical and Laboratory Standards Institute (CLSI), antimicrobial susceptibility test results are categorized into three groups: S (Sensitive): The strain is susceptible to the antibiotic, indicating effective inhibition of bacterial growth at standard dosages. I (Intermediate): The strain demonstrates intermediate resistance to the antibiotic, suggesting potential effectiveness under specific clinical conditions, such as high doses or localized treatment. R (Resistant): The strain is resistant to the antibiotic, meaning the antibiotic is generally ineffective at standard dosages.

Category	Concentration/disc	Interpretation Standard (mm)			Diameter (mm)	Result
		Resistant (R)	Intermediate (I)	Sensitive (S)		
Penicillin	10 U	≤10	11–16	≥17	26.74	S
Vancomycin	30 µg	≤14	15–16	≥17	15.9	I
Oxacillin	1 µg	≤10	11–12	≥13	3	R
Levofloxacin	5 µg	≤13	14–17	≥18	18.26	S
Clindamycin	2 µg	≤14	15–19	≥20	12.1	R
Erythromycin	15 µg	≤13	14–22	≥23	15.22	I
Polymyxin B	300 IU	≤8	9–11	≥12	6.42	R
Gentamicin	10 µg	≤12	13–14	≥15	18.72	S
Lincomycin	2 µg	≤14	15–20	≥21	3	R
Minocycline	30 µg	≤15	16–18	≥19	32.16	S
Tetracycline	30 µg	≤11	12–14	≥15	12.26	I
Chloramphenicol	30 µg	≤12	13–17	≥18	29.56	S
Imipenem	10 µg	≤19	20–22	≥23	17.06	R
Doxycycline	30 µg	≤12	13–15	≥16	17.8	S
Azithromycin	15 µg	≤17	18–19	≥20	25.48	S
Ceftriaxone	30 µg	≤19	20–22	≥23	18.82	R
Ceftazidime	30 µg	≤14	15–17	≥18	19.72	S
Cefoperazone	75 µg	≤22	23–25	≥26	8	R
Ciprofloxacin	5 µg	≤15	16–20	≥21	12	R
Norfloxacin	10 µg	≤12	13–16	≥17	6.44	R
Florfenicol	30 µg	≤12	13–17	≥18	31.56	S
Piperacillin	100 µg	≤17	18–20	≥21	29.32	S
Streptomycin	10 µg	≤6	7–9	≥10	3	R
Compound- Sulfonamides	25 µg	≤10	11–15	≥16	3	R
Ampicillin	10 µg	≤13	14–16	≥17	24.14	S
Kanamycin	30 µg	≤13	14–17	≥18	3	R
Amikacin	30 µg	≤14	15–16	≥17	17.98	S
Cefuroxim	30 µg	≤14	15–17	≥18	18.48	S
Cephalexin	30 µg	≤14	15–17	≥18	22.64	S
Cefamezin	30 µg	≤14	15–17	≥18	24.42	S

**Table 4:** Comparison of sequence characteristics between *Leucobacter* sp. HNU-1 and six other species in the *Leucobacter* genus.

Item	*Leucobacter* sp. HNU-1	*Leucobacter iarius* JCM 14736	*Leucobacter chromiireducens* TAN 31504	*Leucobacter aridicollis* DSM 17380	*Leucobacter coleopterorum* HDW9A	*Leucobacter komagatae* DSM 8803	*Leucobacter luti* RF6
GeneBank assembly	PRJNA1137138	GCA_039530105.1	GCA_016758195.1	GCA_013409595.1	GCA_011382985.1	GCA_006716085.1	GCA_004217175.1
Length of chromosome/bp	3,375,033	3,524,626	3,537,946	3,573,416	3,215,551	3,752,337	3,618,231
GC content/%	70.37	70.57	68.93	67.32	60.32	66.63	69.46
Number of CDSs	3268	3188	3096	3257	2980	3375	3089
Number of rRNAs	9	3	3	9	6	9	5
Number of tRNAs	52	53	54	52	45	50	46
Number of CRISPRS	6	13	1	2	2	2	3

## Data Availability

The datasets generated and analyzed in the current study are available in the WGS and Genbank in the NCBI repository, PRJNA1137138 and PQ664966.

## Supplementary Materials

The supporting information can be downloaded at: https://www.mdpi.com/article/10.3390/ijms26083673/s1.
