# Supplementary material for: Genome Sequencing of the Antibiotic-Resistant Leucobacter sp. HNU-1 and Its Developmental Toxicity in Caenorhabditis elegans
Source: Int J Mol Sci. 2025 Apr 13;26(8):3673. doi: 10.3390/ijms26083673 (PMC12027743; doi:10.3390/ijms26083673)
Supplement: Supplementary file 1 [file ijms-26-03673-s001.zip › Supplementary figure.pdf]

Genome sequencing of an antibiotic-resistant strain of *Leucobacter sp.*, HNU-1 and its developmental toxicity to *Caenorhabditis elegans*

Jiaming Ju[1], Xinhe Lu[1], Ziqing Gao[1], Hongyan Yin[2],

*Shunqing Xu[*3] and Hanzeng Li[*3]*

Author affiliation:

School of Life and Health Sciences[1], Hainan University, Haikou, China

School of Tropical Agriculture and Forestry, Hainan University, Haikou, Hainan, China

School of Environmental Science and Engineering[3], Hainan University, Haikou, China

[*] Corresponding author, e-mail: Shunqing Xu, Xus@hainanu.edu.cn

Hanzeng Li, hanzeng.li@hainanu.edu.cn;

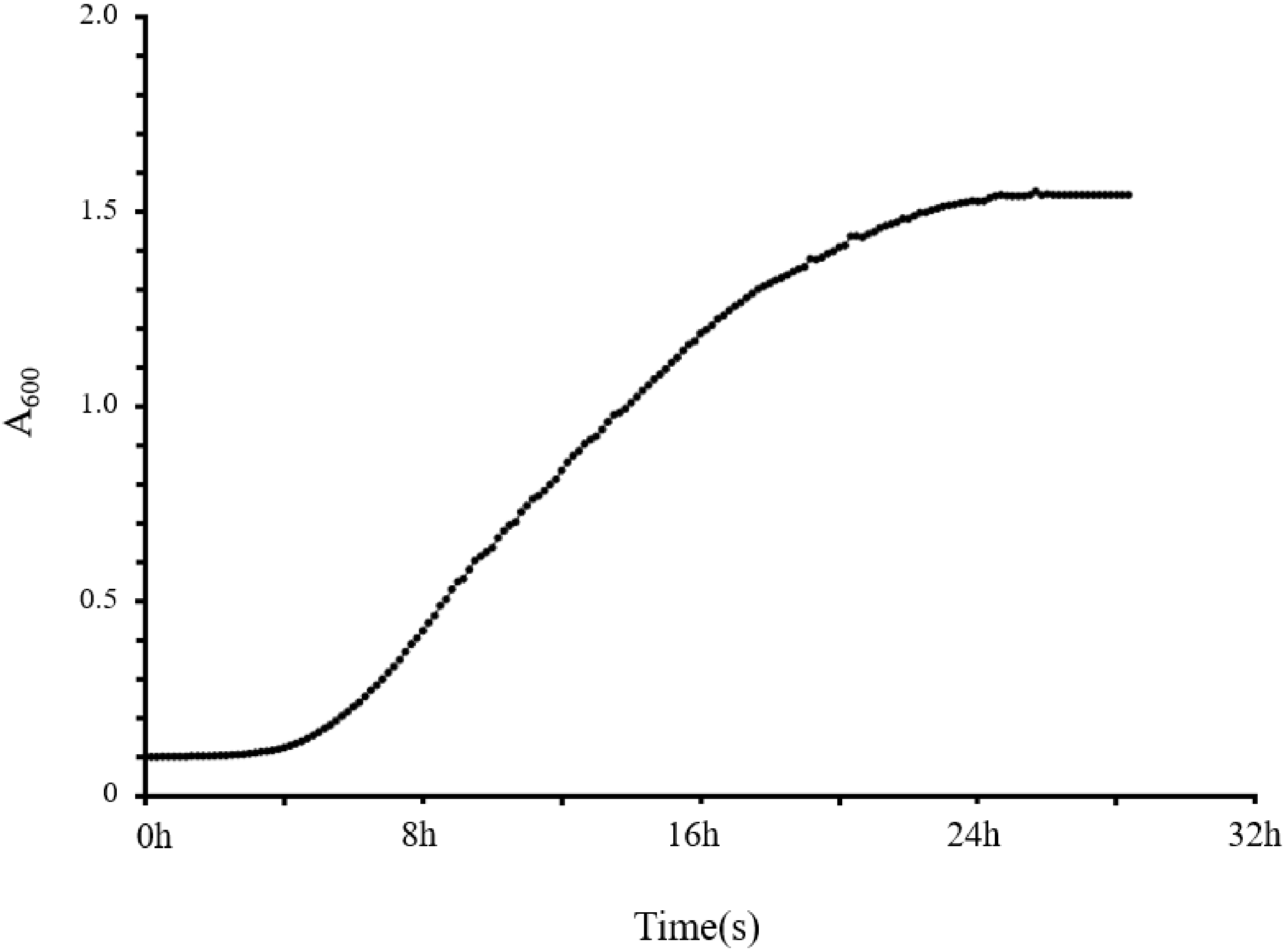

Figure S1. The growth curve of the strain *Leucobacter* sp. HNU-1.

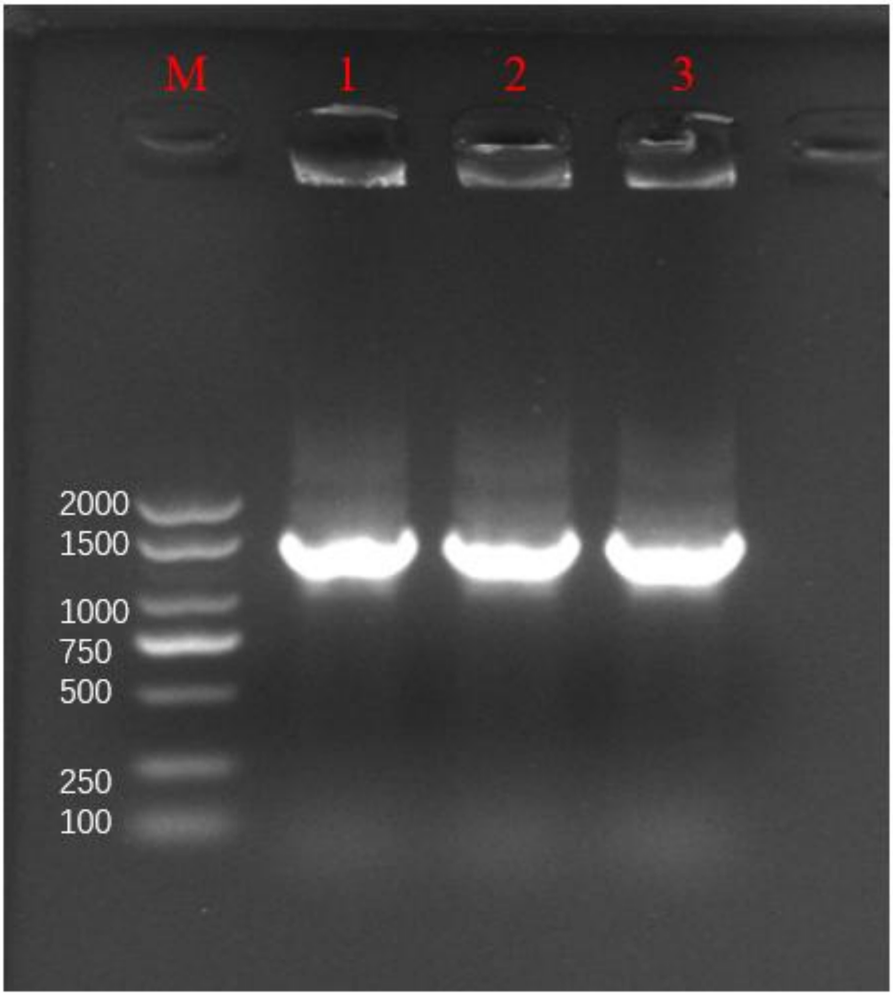

Figure S2. Agarose gel electrophoresis. PCR product of 16S rDNA of *Leucobacter* sp. HNU-1. M(Marker): (DL2000：2000、1000、750、500、250、100bp), 1-3: HNU-1.

```
1     TACGGAGAGT TTGATCCTGG CTCAGGACGA ACGCTGGCGG CGTGCTTAAC ACATGCAAGT
61    CGAACGATGA AGCCCAGCTT GCTGGGTGGA AGAGTGGCGA ACGGGTGAGT AACACGTGAG
121   TAACCTGCCC CGAACTCTGG GATAAGCGCT GGAAACGGCG TCTAATACTG GATATGTCCC
181   ATCACCGCAT GGTGTGTGGG TGGAAAGATT TATCGGTTCG GGATGGACTC GCGGCCTATC
241   AGCTTGTTGG TGAGGTAATG GCTCACCAAG GCGACGACGG GTAGCCGGCC TGAGAGGGTG
301   ACCGGCCACA CTGGGACTGA GACACGGCCC AGACTCCTAC GGGAGGCAGC AGTGGGGAAT
361   ATTGCACAAT GGGCGCAAGC CTGATGCAGC AACGCCGCGT GAGGGATGAC GGCCTTCGGG
421   TTGTAAACCT CTTTTAGTAG GGAAGAAGCG AAAGTGACGG TACCTGCAGA AAAAGCACCG
481   GCTAACTACG TGCCAGCAGC CGCGGTAATA CGTAGGGTGC AAGCGTTGTC CGGAATTATT
541   GGGCGTAAAG AGCTCGTAGG CGGCTTGTCG CGTCTGCTGT GAAAACCCGA GGCTCAACCT
601   CGGGCCTGCA GTGGGTACGG GCAAGCTAGA GTGCGGTAGG GGAGATTGGA ATTCCTGGTG
661   TAGCGGTGGA ATGCGCAGAT ATCAGGAGGA ACACCGATGG CGAAGGCAGA TCTCTGGGCC
721   GTAACTGACG CTGAGGAGCG AAAGCATGGG GAGCGAACAG GATTAGATAC CCTGGTAGTC
781   CATGCCGTAA ACGTTGGGAA CTAGATGTAG GGACTGTTCC ACGGTTTCTG TGTCGTAGCT
841   AACGCATTAA GTTCCCCGCC TGGGGAGTAC GGCCGCAAGG CTAAAACTCA AAGGAATTGA
901   CGGGGGCCCG CACAAGCGGC GGAGCATGCG GATTAATTCG ATGCAACGCG AAGAACCTTA
961   CCAAGGCTTG ACATAGCCGA GAACGCTGTA GAGATACAGA ACTCTTTGGA CACTCGGTTA
1021  CAGGTGGTGC ATGGTTGTCG TCAGCTCGTG TCGTGAGATG TTCGGTTAAG TCCGGCAACG
1081  AGCGCAACCC TCGTCCTATG TTGCCAGCAC GTTATGGTGG GAACTCATGG GATACTGCCG
1141  TGGTCAACAC GGAGGAAGGT GGGGATGACG TCAAATCATC ATGCCCCTTA TGTCTTGGGC
1201  TTCACGCATG CTACAATGGC CGATACAAAG GGCTGCGATA CCGCGAGGTG GAGCGAATCC
1261  CAAAAAGTCG GTCTCAGTTC GGATTGGGGT CTGCAACTCG ACCCCATGAA GTCGGAGTCG
1321  CTAGTAATCG CAGATCAGCA ACGCTGCGGT GAATACGTTC CCGGGCCTTG TACACACCGC
1381  CCGTCAAGTC ATGAAAGTCG GTAACACCCG AAGCCGGTGG CCTAACCTTT TTGGAGGGAG
1441  CCGTCGAAGG TGGGACTGGT GATTAGGACT AAGTCGTAAC AAGGTAGCCG TACCGGAAGG
1501  TGCGGCTGGA TCACCTCCTT T
```

Figure S3. Nucleotide sequence of 16S rDNA from *Leucobacter* sp. HNU-1.
